# Integrated care for multi-domain vulnerability during pregnancy: a retrospective cohort study

**DOI:** 10.1007/s00737-024-01554-x

**Published:** 2025-01-17

**Authors:** Sushma C. Munshi, Eline C. I. Hoex, Anne Marie Weggelaar-Jansen, Esther M. Knijff, Eline C. van der Wilk, Eric A. P. Steegers, Hilmar H. Bijma

**Affiliations:** 1 Erasmus MC – Sophia Children’s Hospital, Department Obstetrics and Gynaecology, Division Obstetrics and Fetal Medicine, University Medical Centre Rotterdam, Dr. Molewaterplein 40, 3015 GD, Rotterdam, South Holland The Netherlands; 2 Tranzo, Tilburg University, Reitse Poort 1, 5000 LE Tilburg, The Netherlands; 3 Department of Psychiatry, Erasmus MC - University Medical Centre Rotterdam, Dr. Molewaterplein 40, 3015 GD Rotterdam, South Holland The Netherlands

**Keywords:** Complex social factors, Substance use, Intellectual disability, Psychiatric illness, Psychosocial care, Integrated approach

## Abstract

**Purpose:**

Psychosocial risk factors are frequently present in pregnant women and are associated with adverse maternal and neonatal outcomes. Professional guidelines recommend early detection of vulnerability and provision of multidisciplinary care, including an integrated care plan for pregnant women with social factors, such as residing in deprived areas, teenage pregnancy, and psychiatric illness. However, to date, such approach is impeded by lack of data on co-occurrence of vulnerability. Therefore, we aim to describe co-occurrence of psychiatric illness, social factors or substance use during pregnancy.

**Methods:**

A retrospective cohort study of 1002 pregnant women referred for evaluation by a multidisciplinary team because of psychiatric illness, social factors or substance use in a university hospital in a large city in the Netherlands. Data from medical charts between January 2017 and May 2022 were extracted and analyzed by univariate and bivariate analysis.

**Results:**

Multi-domain vulnerability was present in 83% of women and most frequently involved the following patterns: psychiatric illness with social factors and chronic physical illness (24%), psychiatric illness with social factors (14%), social factors with chronic physical illness (13%) and psychiatric illness with chronic physical illness (12%). Single-domain vulnerability was present in 17% of women, involving most frequently social factors (9%) and psychiatric illness (8%).

**Conclusion:**

The majority of women with psychiatric illness, social factors or substance use have multi-domain vulnerability. There is a need for a novel approach to care to address vulnerability in pregnant women.

## Introduction

Psychosocial risk factors are present in 36 percent of pregnant women in the UK (Harron et al. 2021). These risk factors include residing in deprived areas, teenage pregnancy, and psychiatric illness (de Graaf et al. 2013a, b; NICE 2010). Psychosocial risk factors are of substantial importance within healthcare due to their association with adverse maternal, fetal and neonatal outcomes. Such outcomes include pre-eclampsia, small for gestational age, preterm birth, as well as unplanned infant admission for injury and infant mortality (Harron et al. 2021; Jones et al. 2022; Knight et al. 2021; Kramer 2015; Mattsson et al. 2022; Pierdant et al. 2023).

Although in the Netherlands no professional guidelines exist, the American College of Obstetricians and Gynaecologists in the USA and the National Institute for Health and Care Excellence in the UK recommend early identification of vulnerability and provision of multidisciplinary care, including an integrated care plan for pregnant women facing social factors, such as domestic abuse, substance use, recent migration, and psychiatric illness (ACOG 2006; NICE 2010, 2014; Kendig et al. 2017). However, the implementation of such integrated care which combines medical, psychiatric, and social care is impeded by siloed health care systems as observed in the UK, USA and the Netherlands (Bos et al. 2021; NHS Confederation 2022; Hoffman 2021; Knight et al. 2021). Data on the implementation of such approach to care and on patterns of multi-domain vulnerability during pregnancy are scarce, as most existing approaches focus on single vulnerabilities, like substance use or psychiatric illness (Flannigan et al. 2023; Lomonaco-Haycraft et al. 2018). Currently, only one study evaluated more than two domains of vulnerability during pregnancy, reporting multi-domain vulnerability in 5,1% of women based on ICD-10 codes, although without specific mention of psychiatric illness and with limited exploration of psychosocial factors (Brembilla et al. 2019). Other studies examined co-occurrence of vulnerability limited to two domains, like social factors and psychiatric illness, or chronic physical illness and psychiatric illness (Barrios et al. 2021; Biaggi et al. 2016; Bouvette-Turcot et al. 2017).

Therefore, the aim of this study is to describe patterns of co-occurrence of vulnerability in multiple domains in pregnant women with psychiatric illness, social factors or substance use by evaluating data derived from an integrated care approach implemented in a tertiary level hospital in the Netherlands.

## Materials and methods

### Participants

**Fig. 1:**
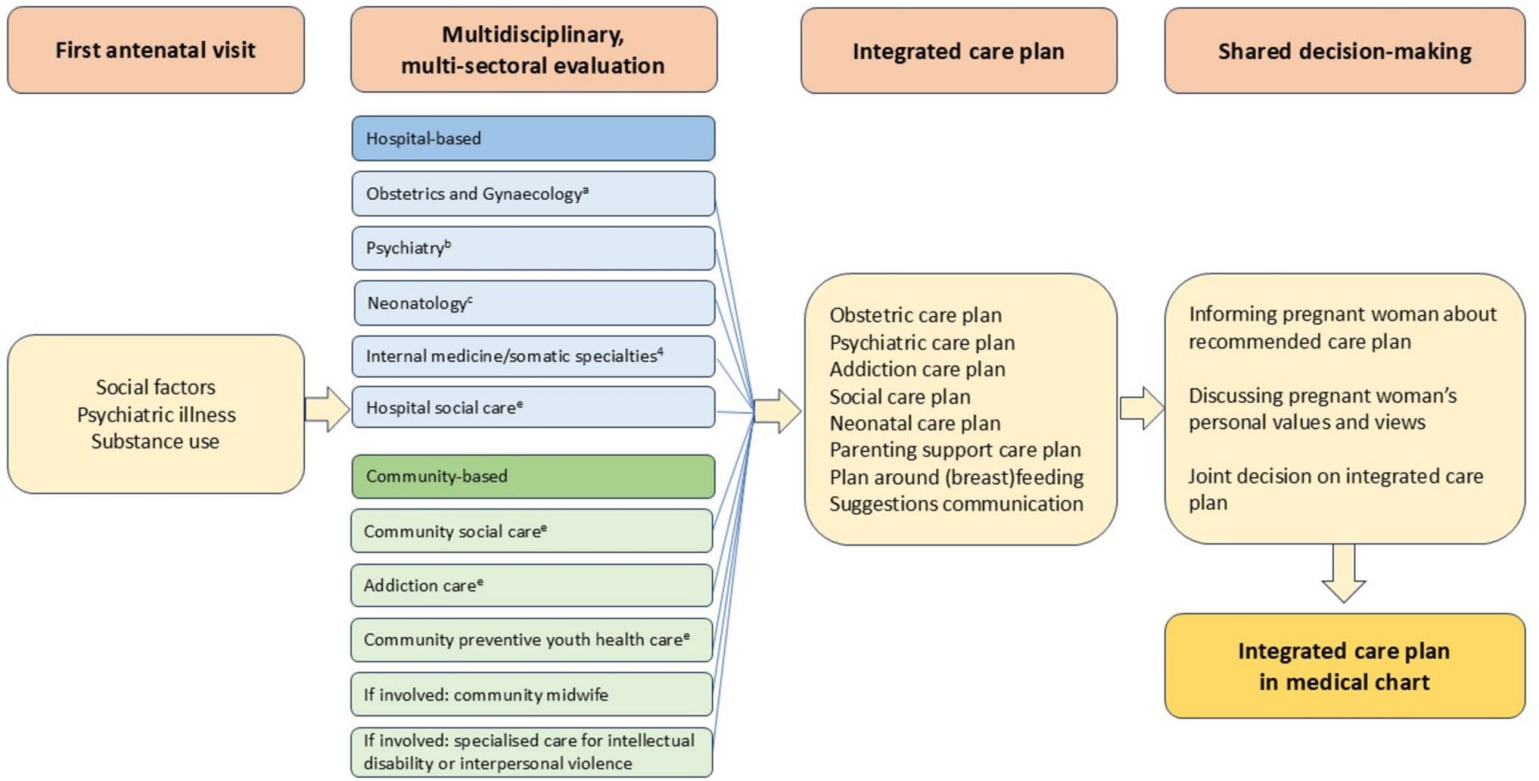
Development of the integrated care plan. ^a^Maternal Fetal Medicine specialist, midwife, obstetric physician assistant, obstetric nurse,^b^Psychiatrist, psychiatric nurse, psychiatric physician assistant, ^c^Neonatal physician assistant, ^d^If involved internal medicine specialist, or physician assistant (e.g. in case of HIV-infection, gestational diabetes or inborn errors of metabolism), ^e^Social worker

We included all pregnant women^1^ who were referred for evaluation by our multidisciplinary team because of psychiatric illness, severe or multiple social factors or substance use (see Table 1). Maternity care in the Netherlands is organized into various levels based on medical risks. Frequently, the first booking is with a community midwife after which women with risk factors are referred to hospital-based obstetric care (van Blarikom et al. 2022). The multidisciplinary team consists of medical, social, and mental health care providers who meet once a week for one hour with the same team (see Fig. 1 for a detailed description of the composition of the team). Pregnant women under the care of the multidisciplinary team between January 2017 and May 2022 were included. All women received obstetric care in the Erasmus MC University Medical Centre in Rotterdam, the second largest city of the Netherlands, with large perinatal health disparities related to socio-economic status and neighbourhood (de Graaf et al. 2013b; Ravelli et al. 2013; Timmermans et al. 2011; Vos et al. 2014). In case of multiple pregnancies, we evaluated only the first pregnancy. All participants were self-identified women.

**Table 1:** Indications for evaluation by multidisciplinary team

Substance use and alcohol misuse:
- Severe substance use (alcohol/illicit drugs) in history
- (Suspected) drug and/or alcohol abuse after positive pregnancy test
- Substance use, cannabis use over 3 times a week and/or binge drinking (> 6 alcohol units in 2 h) after first day of last menstrual period
Psycho-social:
- R4U score (antenatal risk assessment) > 161(Vos et al. 2015)
- Involvement of child protective services
- Unsafe situation for new-born (for example domestic violence, no electricity/housing/nutrition)
- Financial issues leading to lack of basic necessities (electricity/housing/nutrition)
- Lack of prenatal care/late booking (first visit after 20 weeks gestational age)
- Seeking asylum
- Concern about parent–child interaction
- Intention to give the child up for adoption
- Imprisonment of patient or partner
- Maternal stress interfering with treatment or daily life activities
- Multiple social issues (3 or more domains)
Psychiatric:
- (Suspected) psychiatric illness
- History of perinatal psychiatric illness (postpartum depression, anxiety, PTSD or psychosis)
- History of bipolar disorder
- History of eating disorder
- Use of psychotropic medication
(Suspected) intellectual disability:
- IQ < 70
- IQ 70–85 with concomitant problem
- Intellectual disability interfering with medical care
Avoidance of care:
- Unexplained lack of attending antenatal care (missed > = 2 appointments without cancellation)
Other issues:
- Suspected vulnerability by obstetric care providers

### Setting

Since 2017, we have a multidisciplinary approach for pregnant women facing psychiatric illness, social factors or substance use and alcohol misuse. Cases are presented in a standardized format, with integrated information from all relevant domains. Care providers of each domain contribute their perspectives and recommendations for management. Subsequently, the team collaboratively formulates a shared evaluation of the situation and delineates a sequential, integrated plan of action. This plan encompasses strategies for enhancing patient engagement with care and fostering a positive patient-provider relationship. At booking visit, obstetric care providers evaluate psychiatric illness, social factors and substance use and subsequently refer women, with informed consent, for standardized evaluation of domains of risk factors by a multidisciplinary team. Figure 1 shows the way the care for this group of patients is organized. The following domains were evaluated by the team: *1* Somatic vulnerability, including chronic physical illness; *2* Psychiatric illness or use of psychotropic medication; *3* Substance use (including cannabis) and alcohol misuse; *4* Social factors and *5* Intellectual disability. The team consists of representatives of both hospital and community services, such as general and specific community social care and preventive youth care.

### Data collection

We extracted socio-demographic characteristics and data on domains of vulnerability from the medical charts and the integrated care plans. The timeframe submitted for access to pregnant women’s medical charts was 3–6 months and medical charts were only accessed by members of the medical team involved in their care. Chronic physical illness was defined as any physical illness or congenital abnormality that was present before pregnancy and required (or was expected to require) at least six months of ongoing medical attention or limited (or was expected to limit) activities of daily living or both. Psychiatric illness was defined as current psychiatric illness according to Diagnostic and Statistical Manual of Mental Disorders Fifth Edition or current use of psychotropic medication for a psychiatric illness (American Psychiatric Association 2013). Substance use was defined as current substance use disorder or alcohol use disorder according to Diagnostic and Statistical Manual of Mental Disorders Fifth Edition (American Psychiatric Association 2013), except for cannabis use, which was defined as any use of cannabis during pregnancy. Moreover, we considered psychiatric vulnerability present in case of history of a psychiatric illness with relapse during the perinatal period, such as perinatal depression. Social factors were defined as unfavourable relationship, family and/or social support circumstances, unintended pregnancy, financial issues that limited access to daily necessities, severe housing issues, migration, asylum-seeking or refugee status, teenage pregnancy aged under 20 years, involvement of Child Protective Services, and mental health issues without a formal psychiatric diagnosis. Intellectual disability was defined as an IQ below 70 or an IQ below 85 with impact on daily functioning (American Association on Intellectual and Developmental Disabilities, 2021). Additionally, we considered intellectual disability present if a medical doctor (usually a psychiatrist) clinically strongly suspected intellectual disability in women without middle or higher vocational education or if mentioned by women at history taking.

### Statistical analysis

We used descriptive statistics, including frequencies, percentages, means, standard deviations, medians, and ranges for analyzing baseline variables to analyze the data. Co-occurrence of multiple domains was evaluated by cross-tabulation analysis and an UpSet Plot (Lex et al. 2014). The UpSet Plot analyses the frequency of certain co-occurrences and then depicts interactive co-occurrence of multiple variables. Missing values were treated as missing in bivariate analyses and were not imputed. Statistical analyses were performed using the Statistical Package for the Social Sciences (SPSS), version 28.1.0.1 (142) and R*,* version 4.1.3.

## Results

### Participant characteristics

Table 2 shows the characteristics of 1002 pregnant women^1^ who were discussed by the multidisciplinary team. They represent 12,4% of women who gave birth in our hospital during this period of time. The situation of women was on average discussed 2.3 (range 1–12) times. Mean age was 30.9 (sd 6.10) years. In 37,0% of women obstetric history was complicated by previous small for gestational age, preterm birth, congenital abnormality or intrauterine fetal death (IUFD).

**Table 2:** Maternal Characteristics

Maternal Characteristics	*N* = 1002
Age in years, mean (SD)	30.9 (6.10)
Gestational age at booking visit in our hospital, weeks, mean (SD)	19.3 (8.84)
Gestational age at multidisciplinary evaluation, weeks, mean (SD)	23.2 (8.55)
Smoking n, (%)	170 (17.0)
Previous abortion n, (%)^a^	195 (19.8)
Single previous abortion n, (%)	124 (12.6)
Multiple previous abortion n, (%)	71 (7.2)
Multiparous n, (%)^b^	586 (59.0)
Complicated obstetric history (in multiparous women)	217/58 (37.0)
Previous small for gestational age n, (%)^c^	98/587 (16.7)
Previous preterm birth n, (%)^d^	97/587 (16.5)
Previous congenital abnormality n, (%)^e^	45/587 (7.7)
Previous IUFD n, (%)^f^	38/587 (6.5)
Involvement Child Protective Services previous child n, (%)	93 (9.3)
Previous child out-of-home placement n, (%)	56 (5.6)
Previous child under custody of Child Protective Services n, (%)	22 (2.2)
Previous child safety plan and monitoring n, (%)	6 (0.6)
Previous child voluntary out-of-home placement or adoption n, (%)	5 (0.5)

^a^19 missing values

^b^8 missing values

^c^4 missing values, previous small for gestational age was defined as a previous child with a birth weight less than the 10th centile (NHS 2020)

^d^2 missing values, previous small for gestational age was defined as a previous birth before 37 weeks of gestational age (Royal College of Obstetricians and Gynaecologists 2013)

^e^1 missing value

^f^1 missing value

^1^Within this publication, we use the terms pregnant woman and women's health. However, it is important to acknowledge that it is not only people who identify as women for whom it is necessary to access care. Obstetrics and Gynecology services and delivery of care must therefore be appropriate, inclusive and sensitive to the needs of those individuals whose gender identity does not align with the sex they were assigned at birth.

### Domains of vulnerability

Table 3 shows the domains of vulnerability that were evaluated: social factors, psychiatric illness, chronic physical illness, substance use, and intellectual disability.

**Table 3:** Main domains of vulnerability

Main domains of vulnerability	*N* = 1002
Social factors n, (%)	789 (78.7)
Single social factor n, (%)	277 (27.8)
Multiple social factors n, (%)	506 (50.8)
Psychiatric illness n, (%)	658 (65.7)
Single psychiatric illness n, (%)	483 (48.2)
Multiple psychiatric illness n, (%)	175 (17.5)
Chronic physical illness n, (%)	566 (56.5)
Single chronic physical illness n, (%)	355 (35.4)
Multiple chronic physical illness n, (%)	211 (21.1)
Substance use	131 (13.1)
Cannabis use n, (%)	69 (6.9)
Cocaine use n, (%)	47 (4.7)
Alcohol misuse n, (%)	38 (3.8)
Other substance use n, (%)	20 (2.0)
Opiate and benzodiazepine use n, (%)	20 (2.0)
Single substance used n, (%)	268 (26.9)
Multiple substances used n, (%)	112 (11.2)
(Suspected) intellectual disability n, (%)^a^	91 (9.1)

^a^35/91 (38%) of women were identified through a formal assessment of IQ and 56/91 (56%) by a physician

### Social factors

Social factors were present in 78,7% of women: 27,8% women had one and 50,8% women had multiple unfavourable social factors. Most frequent were relationship, family and social support issues (57,0%), severe financial issues (25,6%), and severe housing issues (17,5%) (see Table 4). Pregnancy planning was mentioned in the charts of 680 women. Unintended, desired pregnancy was present in 48,8% of these women and unintended, undesired pregnancy in 4,5%. In 5,2% of women preconception care was used. The use of folic acid was not known for every woman. Teenage pregnancy (< 20 years) was present in 4,5% women. In women with social factors, psychiatric illness was present in 57,5%, chronic physical illness in 55,5%, substance use in 15,6%, and intellectual disability in 11,0% of women.

**Table 4:** Social factors

Social factors	*N* = 1002
Social factors n, (%)	789 (78.7)
Single social factors n, (%)	277 (27.8)
Multiple social factors n, (%)	506 (50.8)
Relationship, family and social support factors n, (%)	571 (57.0)
Insufficient social support network n, (%)^a^	189 (22.5)
Single motherhood n, (%)	175 (17.5)
Severe relationship issues n, (%)	140 (14.0)
Parenting issues previous child n, (%)	132 (13.2)
(Previous) domestic abuse n, (%)^b^	112 (11.2)
Family problems n, (%)	44 (4.4)
Planning pregnancy n, (%)^c^	680 (67.8)
Unintended n, (%)^d^	363 (36.2)
Intended n, (%)	317 (31.6)
Severe financial issues n, (%)	257 (25.6)
Severe debts n, (%)	99 (9.9)
Insufficient money for daily necessities n, (%)	99 (9.9)
No income n, (%)	64 (6.4)
No healthcare insurance n, (%)	26 (2.6)
Housing issues n, (%)	175 (17.5)
Unsuitable housing n, (%)	90 (9.0)
Homeless n, (%)	52 (5.2)
Threatened eviction n, (%)	33 (3.3)
Recent migrant/refugee/asylum seeking/language issues n, (%)	115 (11.5)
Mental health issues without psychiatric diagnosis n, (%)	86 (8.6)
Current involvement Child Protective Services n, (%)	45 (4.5)
Monitoring/safety plan n, (%)	26 (2.6)
Child under custody n, (%)	10 (1.0)
Intended out-of-home placement n, (%)	9 (0.9)
Management unknown n, (%)	6 (0.6)
Teenage pregnancy (< 20 years) n, (%)	45 (4.5)
Other social factors n, (%)^e^	23 (2.3)

^a^161 missing values

^b^(Previous) domestic abuse was defined as an incident of threatening behaviour, violence or abuse (psychological, physical, sexual, financial or emotional) between adults who are or have been intimate partners or family members, regardless of gender or sexuality (NICE 2010)

^c^322 (32.1%) unknown

^d^An unintended pregnancy was defined as a pregnancy that occurred to a women who was not planning to have a child or that it was mistimed, in a sense that it occurred earlier than desired (State of World Population 2022)

^e^Previous imprisonment, prostitution, human trafficking, entry in prenatal care > 20 weeks or no prenatal care

### Psychiatric illness

Psychiatric illness was present in 65,7% of women: 48,2% had one and 17,5% multiple psychiatric diagnoses. The three most frequent diagnoses were depressive disorder (22,0%), anxiety disorder (14,1%), and posttraumatic stress disorder (13,5%) (see Table 5). In 49,5% of women, one or more of these three diagnoses were present. Psychotic disorder was present in 6,7% of women and bipolar disorder in 5,7%. Psychotropic medication during pregnancy was used by 37,9% of women. In women with psychiatric illness, social factors were present in 69,0%, chronic physical illness in 58,8%, substance use in 6,5%, and intellectual disability in 6,5%.

**Table 5:** Psychiatric illness

Psychiatric illness	*N* = 1002
Psychiatric illness n, (%)	658 (65.7)
Single psychiatric illness n, (%)	483 (48.2)
Multiple psychiatric illness n, (%)	175 (17.5)
Use of psychotropic medication n, (%)^a^	380 (37.9)
Single use of psychotropic medication n, (%)	268 (26.7)
Multiple use of psychotropic medication n, (%)	112 (11.2)
*Most used psychotropic medication*	
SSRI n, (%)	171 (17.0)
Benzodiazepine n, (%)	84 (8.4)
Lithium n, (%)	44 (4.4)
Depressive disorder n, (%)	220 (22.0)
Anxiety disorder n, (%)	141 (14.1)
Posttraumatic stress disorder n, (%)	135 (13.5)
Personality disorder n, (%)	122 (12.2)
Borderline personality disorder n, (%)	82 (8.2)
Other personality disorder n, (%)	42 (4.2)
Psychotic disorder n, (%)	67 (6.7)
Unspecified psychotic disorder n, (%)	42 (4.2)
Schizophrenia n, (%)	12 (1.2)
Schizo-affective disorder n, (%)	12 (1.2)
Bipolar disorder n, (%)	57 (5.7)
Attention Deficit (Hyperactivity) Disorder n, (%)^b^	39 (3.9)
Eating disorder n, (%)^c^	27 (2.7)
Previous perinatal psychiatric n, (%)^d^	16 (1.0)
Other psychiatric illnesses n, (%)^e^	19 (1.9)
Autism spectrum disorder n, (%)	14 (1.4)

^a^5 missing values

^b^ADHD, ADD

^c^anorexia nervosa, boulimia, eating disorders unspecified

^d^perinatal depression in history, post-partum psychosis in history, depression remission in history

^e^conversion disorder, adjustment disorder, sleeping disorder, somatic symptom disorder, mophodysmorphic disorder, other specified illness

### Chronic physical illness

Chronic physical illness was present in 56,5% of women: single in 35,4% and multiple in 21,1%. Most frequent chronic physical illnesses were obesity (23,0%), endocrine diseases, such as Polycystic Ovary Syndrome (PCOS), hypothyroidism, and diabetes (11,1%) and auto-immune diseases (8,7%) (see Table 6). When obesity was not included as chronic physical illness, chronic physical illness was present in 433 women (43,2%): single in 32,9% and multiple in 10,3%. In women with chronic physical illness, social factors were present in 77,4%, psychiatric illness in 68,4%, substance use in 7,4% and intellectual disability in 7,4%.

**Table 6:** Chronic physical illness

Chronic physical illness	*N* = 1002
Chronic physical illness n, (%)	566 (56.5)
Single chronic physical illness n, (%)	355 (35.4)
Multiple chronic physical illness n, (%)	211 (21.1)
Obesity (BMI > 30) n, (%)	230 (23.0)
Endocrine diseases n, (%)	113 (11.3)
Poly Cystic Ovary Syndrome n, (%)	52 (5.2)
Hypothyroidism n, (%)	32 (3.2)
Diabetes n, (%)	27 (2.7)
Other endocrine diseases n, (%)	13 (1.3)
Auto-immune diseases n, (%)	87 (8.7)
Endocrine auto-immune disease n, (%)	28 (2.8)
Musculoskeletal auto-immune disease n, (%)	27 (2.7)
Inflammatory bowel disease n, (%)	25 (2.5)
Other auto-immune diseases n, (%)	12 (1.2)
Cardiac-vascular diseases n, (%)	84 (8.4)
Pre-existing hypertension n, (%)	44 (4.4)
Congenital cardiac abnormality n, (%)	23 (2.3)
Rhythm disorders n, (%)	16 (1.6)
Other cardiac-vascular diseases n, (%)	7 (0.7)
Respiratory diseases n, (%)	80 (8.0)
Asthma n, (%)	78 (7.8)
OSAS n, (%)	2 (0.2)
Neurological diseases n, (%)	52 (4.8)
Migraine n, (%)	19 (1.9)
Other neurological diseases n, (%)^a^	33 (3.3)
Haematological diseases n, (%)^b^	36 (3.6)
Gastro-intestinal diseases n, (%)	26 (2.6)
Gastric bypass n, (%)	20 (2.0)
Gastric sleeve n, (%)	4 (0.4)
Other gastro-intestinal diseases n, (%)	2 (0.2)
Medically unexplained somatic symptoms n, (%)	27 (2.7)
Chronic infection n, (%)^c^	23 (2.3)
Genetic syndrome/congenital disorder n, (%)^d^	22 (2.2)
Gynaecological disorder n, (%)	19 (1.9)
Endometriosis n, (%)	16 (1.6)
Cervical dysplasia n, (%)	3 (0.3)
Previous malignancy n, (%)	10 (1.0)
Other chronic physical illnesses n, (%)^e^	7 (0.7)

^a^migraine, epilepsy, hernia, MS, myasthemia gravis, tetraplegy, cadau syndrome, narcolepsy, urge to pee, cerebellar ataxia, bleeding from artero venuous malformation, resection of meningioma, idiopathic intracranacial hypertension, cerebral hemorrhage

^b^thrombo Embolism, bleeding disorder, hemoglobinopathy

^c^hepatitis B, hepatitis C, HIV, livercirrhosis, focal nodular hyperplasia, adenoma

^d^Hirschsprung’s disease, stickler syndrome, paramyotonia congenital, goldenhar syndrome, tuberous sclerosis, multiple epifysaire, triple X syndrome, kartagener syndrome, vascular malformation epiglottis, unilateral renal agenesia, becker myotonia, renal abnormality, vascular abnormality, congenital metabolic disorder, duplicate renal system, turner mosaicism, periodic paralysis, 16p11 duplicate, duodena atresia, hearing disability, syndrome of Sphritzen, PSMA, muscle becker mytonia

^e^renal disorders, chronic pain syndrome

### Substance use

Substance use was present in 13,1% of women (see Table 3). Most frequent were cannabis use (6,9%), cocaine use (4,7%), and alcohol use (3,8%). In women with substance use, social factors were present in 93,9%, psychiatric illness in 32,8%, chronic physical illness in 32,1%, and intellectual disability in 14,5%.

### Intellectual disability

Intellectual disability was present in 9,1% of women (see Table 3). In women with intellectual disability, social factors were present in 95,6%, psychiatric illness in 47,3%, chronic physical illness in 46,2%, and substance use in 20,9%.

### Trauma and abuse

Table 7 shows data on trauma and abuse. Remarkably, even though trauma and abuse were not routinely screened for, 41,3% of all women were known with current or previous adult abuse or had a history of childhood abuse or neglect.

**Table 7:** Abuse and trauma

Abuse and trauma^a^	*N* = 1002
(Known) abuse and trauma n, (%)	414 (41.3)
(Known) abuse or neglect n, (%)	382 (38.1)
Child only n, (%)	177 (17.7)
Adult only n, (%)	147 (14.7)
Both adult and child n, (%)	58 (5.8)
Unknown period of abuse n, (%)	42 (4.2)
Trauma n, (%)	138 (13.8)
Posttraumatic stress disorder n, (%)	126 (12.6)
Other trauma-related disorders n, (%)	12 (1.2)

^a^History of childhood (sexual) abuse or neglect, forced prostitution, adult (sexual) abuse, human trafficking, the presence of posttraumatic stress disorder (PTSD) or another trauma-related disorder

### Patterns of vulnerability

**Fig. 2:**
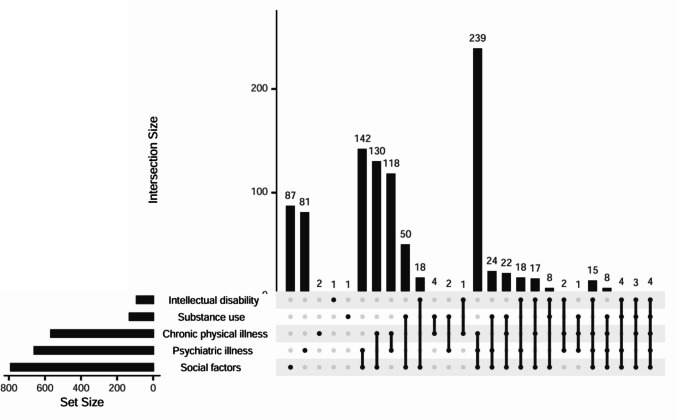
The Upset Plot: Interacting domains of social factors, psychiatric illness, chronic physical illness, substance use and intellectual disability. The connected dots below show patterns of co-occurrence. The bars show the number of women with that pattern of occurrence

Figure 2 shows an Upset Plot depicting the patterns of vulnerability of the five different domains. In 17,2% of women, vulnerability was present in one domain (see Table 8). In case of a single domain, social factors (8,7%) and psychiatric illness (8,1%) were the most common vulnerabilities. In 82,8% of women, multiple domain vulnerability was present. In 46,4% of women vulnerability was present in two domains, in 33,1% in three domains, and in 3,4% in four or five domains.

**Table 8:** Co-occurring domains of vulnerability

Co-occurring domains of vulnerability	*N* = 1002
Single n, (%)	172 (17.2)
Double n, (%)	465 (46.4)
Triple n, (%)	331 (33.1)
Quadruple n, (%)	30 (3.0)
Quintuple n, (%)	4 (0.4)
Patterns of vulnerability n, (%)^a^	
Psychiatric illness, social factors, chronic physical illness n, (%)	239 (23.9)
Psychiatric illness, social factors n, (%)	142 (14.2)
Social factors, chronic physical illness n, (%)	130 (13.0)
Psychiatric illness, chronic physical illness n, (%)	118 (11.8)
Substance use, social factors n, (%)	50 (5.0)

^a^Patterns that were present in less than 5% of the women were not described in this table

When multi-domain vulnerability occurred (82,8%), most frequently involved patterns were psychiatric illness with social factors and chronic physical illness (23,9%), psychiatric illness with social factors (14,2%), social factors with chronic physical illness (13,0%), and psychiatric illness with chronic physical illness (11,8%). Furthermore, in 5,0% of women substance use with social factors was present.

When vulnerability occurred in two domains, the most common patterns were social factors with psychiatric illness (14,2%), social factors with chronic physical illness (13,0%), or psychiatric illness with chronic physical illness (11,8%). When vulnerability occurred in three domains, the most common pattern was psychiatric illness, social factors, and chronic physical illness (23,9%). When vulnerability occurred in four domains, the most common pattern was social factors, psychiatric illness, chronic physical illness, and intellectual disability (1,5%).

**Fig. 3:**
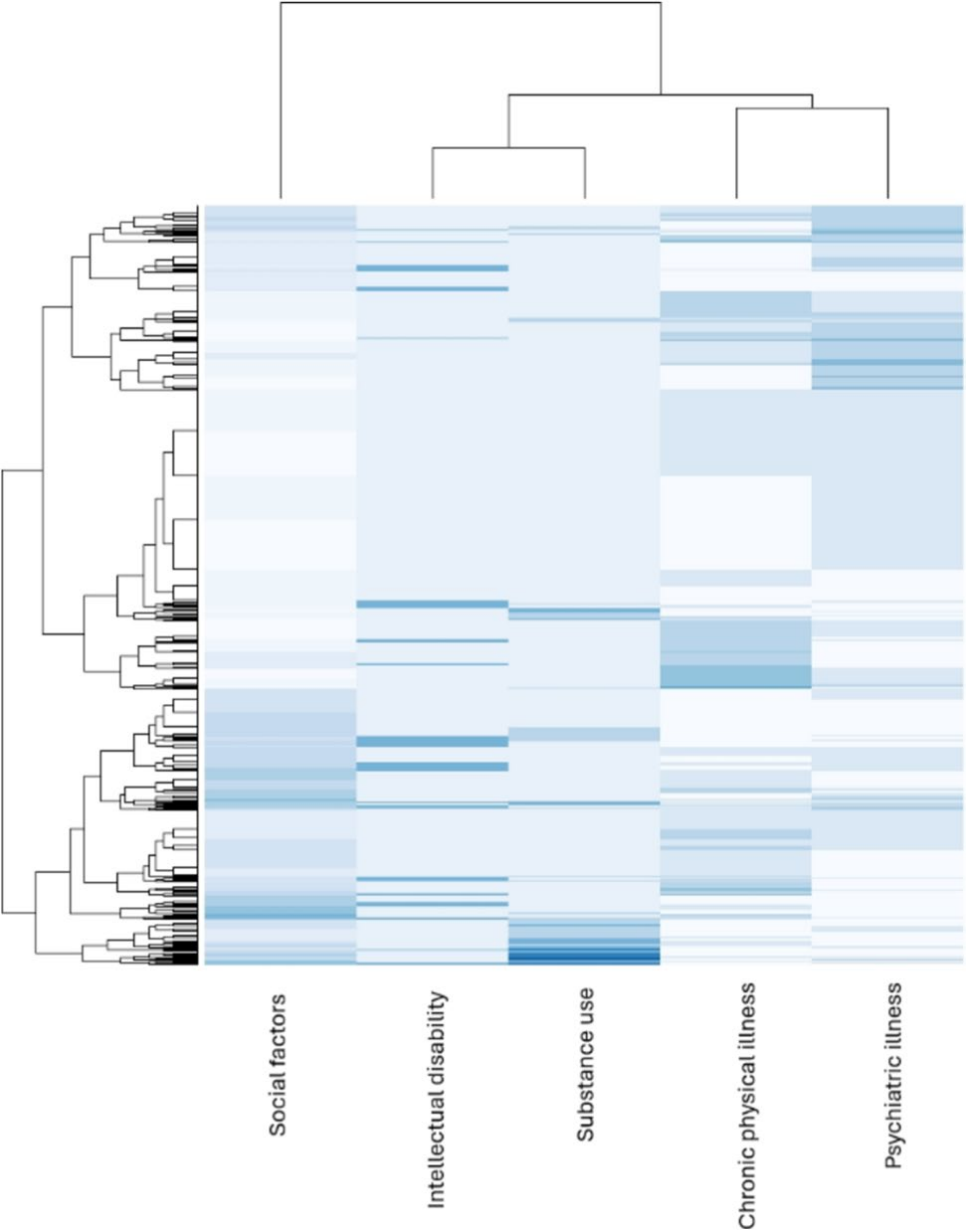
Heat map of the number of vulnerabilities per domain for all participants: Interacting domains of social factors, psychiatric illness, chronic physical illness, substance use and intellectual disability. The colour grading is used to depict the relative number of reported vulnerabilities per domain, with darker blue indicating more co-occurrence of vulnerabilities

Figure 3 shows a heat map illustrating the extent of overlap between the different domains of psychosocial risk factors.

## Discussion

### Principal findings

This study showed multi-domain vulnerability was present in over 80 percent of the women with psychiatric illness, social factors and substance use and most commonly involved a combination of psychiatric illness, social factors and chronic physical illness. Furthermore, chronic physical illness was present in over half of the women and in 37% of the multiparous women obstetric history was complicated by small for gestational age, preterm birth, congenital abnormality or intra-uterine fetal death. Thus, this study contributes highly novel data on multi-domain vulnerability based on data from an integrated care approach that was implemented in our hospital.

### Clinical implications

The frequent co-occurrence of psychiatric illness, social factors and chronic physical illness has several implications for clinical practice. Firstly, it shows the importance of implementing novel integrated care approaches in addressing vulnerability in obstetric care, in line with current guidelines (NICE 2010, 2014). As the most common pattern of co-occurrence comprises psychiatric illness, social factors and chronic physical illness, a pragmatic approach to collaborative care would be to start by fostering collaboration between a psychiatrist, a social worker, and an obstetrician. This multidisciplinary approach serves not only to decide on management and integrated care plans, but also for conveying a cohesive message to patients. Moreover, it fosters enhanced communication among the involved disciplines, facilitating the coordination of services to improve patient care, exchange of domain-specific knowledge, and a more comprehensive alignment of perspectives (Coupland et al. 2021). However, the implementation of integrated care approaches has proven to be challenging, both in obstetrics and other medical fields. Barriers to implementation may arise at the patient, care-provider, and system levels, encompassing issues such as excessive administrative time, high cost, insufficient reimbursement, excessive caseload, inadequate attendance, and lack of leadership (Barrios et al. 2022; Coupland et al. 2021; Schmidt et al. 2023). While various approaches have been proposed for addressing specific adversities, such as substance use (Flannigan et al. 2023), this study represents the first approach to outline multidisciplinary care for multi-domain vulnerability during pregnancy. In our hospital, the feasibility of implementing integrated care for vulnerability was achieved through the establishment of weekly multidisciplinary meetings, following a standardized format in which cases were discussed. During these meetings, it became evident how gaps in knowledge regarding care provision in other domains could impact individual cases and how collaborative care enhanced efficiency and tailored patient care. Consequently, the initial barriers related to time investment and involvement of care providers were mitigated as the benefits of collaboration became apparent. In the future, further steps for clinical implications are to implement similar care approaches in diverse settings and evaluate the impact on obstetric outcomes.

### Research implications

This study has several implications for research. Firstly, to our knowledge, this is the first clinical study that shows multi-domain vulnerability is common in case of social factors and psychiatric illness and that this is frequently accompanied by chronic physical illness. Future research is needed to further elucidate the association of these patterns with obstetric and neonatal outcomes as well as causal relationships in which vulnerability impacts maternal and neonatal outcome. This includes exploring the role of maternal history of early life stress. Studies evaluating the impact of adverse childhood events (ACEs) on pregnancy outcomes show an increased risk of preterm birth, reduced birth weight, and hypertensive disorders of pregnancy in women with a history of ACEs (Miller et al. 2021; Smith et al. 2016). Notably, all three domains within the most common pattern – psychiatric illness, social factors, and chronic physical illness—are recognized for their long-term outcomes stemming from severe early life stress. The seemingly disparate pattern of psychiatric illness, social factors, and chronic physical illness may reflect long-term complications of maternal early life stress, which would have implications and provide guidance for clinical practice. Secondly, future research should aim to explore multiple factors of vulnerability concurrently moving beyond the current focus on single factors. This shift will enable comprehensive understanding of vulnerability patterns and their impact on fetal and neonatal outcomes.

### Strengths and limitations

An important strength of this study is the novel clinical cohort data about patterns of multi-domain vulnerability during pregnancy and as such elucidates a much understudied area of perinatal care, contributing to further development of obstetric practice. Secondly, this study provides an example of how integrated care in obstetric practice is both needed and feasible.

This study also has several limitations. Firstly, retrospective data collection resulted in limited data on unplanned pregnancy, history of trauma or timing of initiation of prenatal care. Secondly, data were collected in a tertiary centre, which limits generalisability of the prevalence of multi-domain vulnerability to pregnant women receiving midwife-led care. Moreover, evaluating associations was challenging due to the non-exclusivity of domains among most pregnant women with multi-domain vulnerability, posing difficulties in constructing a stable regression model. Additionally, given data collection from a tertiary hospital setting, the increased prevalence of social and psychiatric vulnerabilities may potentially result in underestimation of odd ratios. Thirdly, within this study, we adopted a medical model for evaluating vulnerability. It is important to note that the conditions described are not conflated with vulnerability. Rather, vulnerability emanates from a complex interplay of factors, including lack of societal support, systemic barriers, discrimination, inadequate accommodation, and marginalization. (Mitra et al. 2022). As such, we advocate for systemic reforms to address underlying factors to foster inclusivity, empower affected individuals, and promote a holistic approach to health within society.

## Conclusion

Multi-domain vulnerability is present in over 80% of 1002 pregnant women evaluated by a multidisciplinary team because of psychiatric illness, social factors or substance use. The most frequent pattern of vulnerability involved psychiatric illness, social factors, and chronic physical illness (24%). This study shows both the need for and feasibility of novel care approaches addressing vulnerability.
